# Determination of antibiotic consumption index for animal originated foods produced in animal husbandry in Iran, 2010

**DOI:** 10.1186/2052-336X-12-42

**Published:** 2014-01-27

**Authors:** Fathollah Aalipour, Maryam Mirlohi, Mohammd Jalali

**Affiliations:** 1 Food Security Research Center, Department of Food Technology, School of Nutrition and Food Science, Isfahan University of Medical Science, Hezargrib Street, Isfahan, Iran

**Keywords:** Antibiotic, Animal farm, Food, Exposure, Iran

## Abstract

The public health concerns over the long-term exposure to antibiotics have risen in different parts of the world. The purpose of this study was to investigate the antibiotic consumption pattern in livestock and poultry and to estimate the quantity of antibiotic active ingredient (mg) consumed per unit weight (Kg) of red meat, milk and egg production in Iran in 2010. A cross-sectional study was designed in charmahal - bakhtiary province-Iran. A questioner has been developed by naming 110 types of antibiotics. Twenty two veterinary clinicians and three livestock pharmaceutical distributor companies were included in the survey to determine the antibiotic prescription and distribution pattern in the farms. Veterinary organization of Iran supplied the information of the total antibiotic consumption in different dosage forms. National and international data on the livestock and poultry production were obtained from the relevant official web sites. Tetracycline class of antibiotics was the most common types of antibacterial prescribed and sold to both livestock and poultry farms. Amino glycoside, penicillin and macrolide in the cattle farms and furofenocole in broiler farms were the second most used groups of antibiotics. The quantity of antibiotic active ingredients consumed per unit weight of animal-originated food products was counted as 107.4 mg/kg for both milk and red meat and 249.5 mg/kg for broiler meat and egg. Totally, it was estimated that 133 mg antibiotic substances was used per kg of milk, meat and egg produced in 2010. In comparison to available data for other countries, consumption of antibiotics in livestock and poultry in Iran is higher than developed countries with an exception of South Korea. The findings of the present study could be alarming for the legislative authorities in food security and safety. More clear evaluation should be carried out as well as implementation of national monitoring and inspective programs in order to reach an added safety regarding animal-originated foods.

## Introduction

Antibiotics are the most important group of anti-microbial drugs, widely prescribed for human, and animals. It is estimated that 100–200 thousand tons of antibiotic substances are annually produced in the world [1,2]. According to World Health Organization (WHO), about half of the worldwide-produced antibiotics are consumed for non-human applications [3]. Veterinary use of antibiotics was partially specified for prophylactic and growth promotion purposes. Uncontrolled use of antibiotics in animals leaves some residues in meat, milk and egg, which could be harmful to humans. In other words, development of antibiotic resistant bacteria and allergic reactions in humans is known as the consequences of long term ingestion of antibiotics [4]. European Union (EU), Food and Agriculture Organization (FAO) and Food and Drug Administration (FDA) have established certain regulations to monitor the antibiotic residues in foods of animal origins [5]. For instance, in Euro zone, In order to reduce the human exposure to antibiotic residues, the consumption of any antibiotic is prohibited for growth promotion purposes [6]. However, the evaluation of the exposure to antibiotic residues has always been controversial particularly, in the geographical regions where do not follow the restrictive regulations on the use of veterinary antibiotics. In this regard, many attempts have been made to determine the antibiotic residues in animal-originated foods [7-9].

Exposure evaluation may also been performed using a deterministic approach where, in a certain society, the weight ratio of the total consumed antibiotics to the produced animal-originated foods is defined as an antibiotic consumption index [10,11]. For instance, the antibiotic consumption index was previously reported to be 26 and 100 mg/Kg in animal products in Australia and the United State of America, respectively [10,12].

In Iran, there is some evidence of violation in antibiotic residues among the tested food samples [13-16].

However, no study has been conducted to demonstrate the exposure to antibiotics through food consumption. Moreover, there is no recorded report on the diversity of antibiotics consumption in livestock and poultry farms in Iran.

The objectives of this study were to characterize the antibiotic utilization pattern in Iran livestock and poultry farms. In addition, the antibiotic consumption index concerning animal-originated food produced in the year of 2010 was also investigated.

## Materials and method

### Study design

This survey was a cross-sectional descriptive study conducted in the Charmahal- Bakhtiary province in Iran. This province is considered as one of the major livestock production regions in Iran. The study was designed in three parts as following: Firstly, the prescription rate of antibiotics for livestock and broiler farms surveyed through a face-to-face interview by veterinary clinicians. Secondly, the distribution pattern of the sold antibiotics in the tested area was determined by some interviews with animal pharmaceutical distributors. Finally, the antibiotic consumption index was calculated by using the data obtained from official veterinary and agriculture authorities in Iran.

### Investigation of the prescription rate and the distribution pattern of different antibiotics

Two types of questionnaire were prepared in order to determine the prescription rate and distribution pattern of antibiotics, each contained 110 antibiotics in 10 major class including penicillins, tetracyclines, cephalosporins, macrolides, amino-glycosides, aminocyclitol, sulfonamides, nitrofourans, fluoroquinolones and fungicides, all are usable in veterinary practicing [17,18]. The veterinary clinicians (n = 22) were asked to determine the prescription frequency of the given antibiotics in the questionnaire by scoring from one to five (zero = never, one = very low, two = low, three = moderate, four = high and five = very high). The distribution pattern of the sold antibiotics was determined using the information supplied by three main animal pharmaceutical distributer companies in the investigated area. They were asked to clearly determine the amount, type and the form of commercial antibiotics sold to livestock and poultry farms in 2010. Accredited veterinary references [17,18] were used to determine the exact amounts of active ingredients, implemented in to the distributed commercial products. Volume percentage of each class of antibiotics being sold by interviewed companies was expressed as the consumption percentage.

### Determination of antibiotic consumption index

The Veterinary Organization of Iran was requested to provide the data on the total amount of antibiotics used on anmals in 2010 in the country. Since the given information was presented as the number of unit packages in four different dosage forms of antibiotics (Injectable, non-injectable, sachet and solution), the amount of active ingredient for each dosage form was calculated using the following equation [18].

(1) $$E=n\times \sum \left(C\times V\right)/N\times 10^{3}$$

Where *E*: The total amount of antibiotic active ingredient for each dosage form (kg), *n*: the number of each packaged antibiotic dosage form presented by the veterinary Organization of Iran *C*: the concentration of antibiotic active ingredient (%) for each type of antibiotic, included in each dosage form, *V*: the net weight or volume of the package (g or ml), *N*: the number of antibiotic types that were offered through the given dosage form and *10*^*3*^ is a conversion coefficient of g to kg.

The total amount of utilized antibiotic active ingredient supplied by four dosage forms was calculated using the Equation no. 2*:*

(2) $$E_{t}=E_{\text{Injectable}}+E_{\text{non}-\text{Injectable}}+E_{\text{sachet}}+E_{\text{solution}}$$

Where *E*_*t*_ is the total amount of antibiotic active ingredient consumed in Iran in 2010 (kg).

Information on the animal originated food production was obtained from the ministry of agriculture’s official website [19]. Data on animal origin foods for other countries was derived from food and agriculture organization report [20].

Antibiotic consumption index was considered as an exposure calculated as proportion of the quantity of the total antibiotic active ingredient (mg) consumed per unit weight (Kg) of animal originated food produced in 2010 index [10,21] which was calculated using the following equation:

(3) $$\begin{array}{l} \text{Antibiotic}\;\text{consumption}\;\text{factor}\;=\;\text{The}\;\text{annual}\;\text{amount}\;\text{of}\\ \;\text{antibiotic}\;\text{active}\;\text{ingredient}\;\text{used}\;\mathrm{on}\;\text{animals}\;\left(\mathrm{mg}\right)/\\ \;\text{the}\;\text{weight}\;\text{of}\;\text{food}\;\text{animal}\;\text{produced}\;\text{annually}\;\left(\text{Kg}\right) \end{array}$$

### Statistical analysis

Data analysis was performed using Microsoft Office Excel version 2003.

## Result

In Table 1 selected antibiotics based on their prescribed rate, scored by the veterinary clinicians is presented. To simplify the results, five previously- mentioned groups of antibiotics were briefed into three groups for both livestock and poultry farms. Among the high-consumed antibiotics, tetracycline, sulfadiazine and their derivatives were prevalent.

**Table 1:** Selection of antibiotics based on their prescription rate by veterinary clinicians

**Livestock**			**Poultry**		
^**a**^**Low**	^**b**^**Medium**	^**c**^**High**	**Low**	**Medium**	**High**
Erythromycin	Tilmycozin	Oxytetracyclin	Tylosin	Chlortetracyclin	Oxytetracyclin
Doxycycline	Flurofenocol	Gentamycin	Flumequine	Lincomycin	Florfenicol
Penicillin v	Kanamycin	Tylosin	Tiamolin	Tetracycline	Sulfadiazine
Ceftriaxone	Penicillin k, Na	Sulfadiazine	Sulfametazin	Colistin	Doxycycline
Sufametazole	Dehydrostreptomycin	Lincomycin	Neomycin	Enrofloxacin	Erythromycin
Myconazole	Sulfametazin	Cotrimosazole	Spectinomycin	Cotrimosazole	Tremetoprim
Ceftiofur	Neomycin	Streptomycin	Sulfametoksacin		
Ketoconazole	Ampicillin	Benzathin	Bacitracin		
Katkabod	Chlortetracyclin	penicillin	Tilmycozin		
Bacitracin	Enrofloxacin	Tetracycline	Gentamycin		
Nistatin	Sulfametoksacin	Tremetoprim	Kanamycin		
Cloxacillin	Amoxicillin	Pencillin	Amoxicillin		
Cephalexin	Colistin	procaine	Kitazamycin		
Nitroforazonlidon		Spectinomycin	Chloramphenicole		
Chlotrimazole					
Klindamycin					
Flumequine					

The results of 5 score questionnaire were briefed to 3 groups; a) low (seldom prescribed: 1, 2), b) medium (occasionally prescribed: 3) and c) high (frequently prescribed: 4, 5).

The results of the questionnaires filled out by pharmaceutical distributor companies showed that 48 out of 110 questioned types of antibiotics have been frequently sold to the animal farms in 2010.

**Figure 1:**
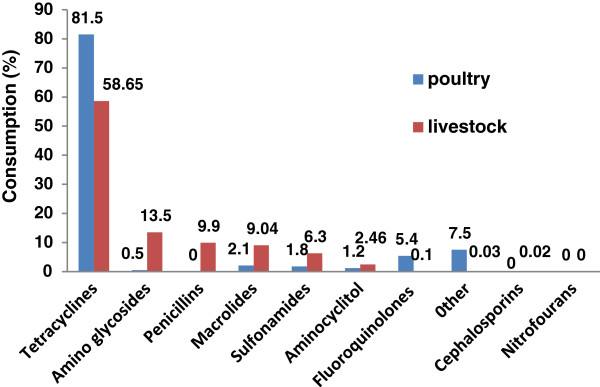
Distribution pattern of antibiotic sold out by interviewed veterinary pharmaceutical companies.

The distribution pattern of antibiotics sold out by interviewed veterinary pharmaceutical companies is illustrated in Figure 1. Tetracycline class of antibiotics was the most common antibiotics sold for both livestock and poultry farms. In livestock farms 90% of the total distributed commercial antibiotics composed of tetracycline, amino glycosides, penicillin and macrolied, whereas, in poultry farms 81% of the total sold antibiotics was estimated to be only tetracycline. None of the prohibited antibiotics such as chloramphenicol and nitrofouran group was traded as stated by interviewed companies.

**Figure 2:**
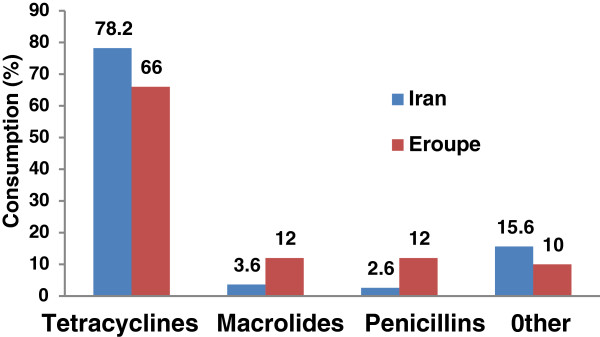
Antibiotic consumption pattern in the studied area and Euro zone.

Figure 2 shows the consumption pattern of three major classes of antibiotics investigated in this study compared with that of European countries. As shown, in the investigated farms, the consumption of penicillin and macrolied commonly used for therapeutic purpose is less than that of Euro zone. In contrast, the consumption of broad-spectrum antibiotic tetracycline mainly used for therapeutic, prophylactic and growth promotion purpose is significantly higher in the surveyed aria.

Table 2, Presents the total volume of antibiotics used in animal farms of Iran in 2010. About 1200 and 607 tons of antibiotics for cattle and poultry were consumed, respectively.

**Table 2:** The total volume of antibiotics distributed in animal farms in Iran in 2010

**Farms**	**Dosage form of antibiotic**	^**a**^**Number of unite packaging**	**Mean weight of the packages (g)**	**Average concentration of the active ingredient (%)**	**Total active ingredient (kg)**	**Total (ton)**
**Livestock**	Injectable	16970196 (Vial)	87.7	19.1	284262.66	1199.689
	^b^Non Injectable	100951622 (Pack)	26.284	34.50	915427 .28	
**Poultry**	Water soluble	1958225 (liter)	-	16.4	321148	607.206
	Sachet	1030530 (Pack)	1920.1	14.45	286059	
**Total**						1806.896

^a^Information was provided by veterinary organization of Iran.

^b^Included different forms; soluble, pills, powder and bolus.

In Table 3 the entire animals originated food production data in Iran in 2010 is tabulated. About 13889000 tons of animals originated food products were supplied in 2010 of which 2% was constituted by aquatic, honey and fowls other than chicken. Moreover, livestock products had the highest share, about 80% of the total products.

**Table 3:** **Animal food production in Iran in 2010**^**ab**^

	**Fowl meat other than chicken**	**Honey**	**Aquatic cultural**	**Egg**	**Chicken meat**	**Milk**	**Red meat**
Production	21	45	214	766.7	1666.5	10242	933.6
Volume percentage	0.2	0.3	1.5	5.5	12	73.7	6.7

^a^Information was adapted from agriculture ministry of Iran.

^b^Presented data is expressed by the unit of 1000 tons.

According to Tables 2 and 3, antibiotic consumption factor was revealed 107.3 mg per kg for production of red meat and milk, and 249.5 mg per kg for production of chicken and egg. Meat, milk and egg annual production data of several countries comparing to Iran is shown in Table 4.

**Table 4:** **Livestock and poultry production data in different countries**^**a**^

**Country**	**Meat**	**Milk**	**Egg**	**Total production**	**Year**	**Reference**
Iran	2600	10242	766.7	13609	2010	This study
Ireland	982	5200	33	6215	2007	[20]
France	5062	24549	765	30375	2007	[20]
Denmark	2061	4600	78	6739	2007	[20]
Germany	7053	27935	800	35788	2007	[20]
British	3411	14450	608	18469	2007	[20]
Austria	4164	10350	166	14680	2007	[20]
Korea	1754	2145	574	4473	2007	[20]
USA^b^	37839	77314	4863	120016	1995-2007	[20]
European Union	45132	169061	6883	221076	2007	[20]

^a^Presented data is expressed by the unit of 1000 tons.

^b^Data is an average of the annual production between 1995–2007.

Using the official representative data, the overall production of meat, milk and egg in France, United state of America and Europe in 2007 was counted to be 2.2, 8.8 and 16.2 times more than that of Iran in 2010, respectively.

Table 5 shows the antibiotic utilization data as well as antibiotic consumption index in the husbandry sector of different countries.

**Table 5:** Total amount of utilized antibiotics and antibiotic consumption index in diferent countries

**Country**	**Weight of antibiotics utilized in the farms (Tons)**	**Antibiotic active ingredients consumed per one kg meat (mg/kg/year)**	**Antibiotic consumed per one kg of animal products (mg/kg/year)**^**a**^	**Reference**
				
Iran	1806	694.9	132.8	This study
Ireland	104	106	17	[22]
France	1320	261	43	[21]
Denmark	111	53.9	16.5	[23]
Germany	668.8	94.8	18.7	[23]
British	414	121.4	22.4	[23]
Austria	113	27.1	7.7	[23]
Korea	1,278	728.6	285.7	[24]
USA	11340	300	94	[1]
European Union	4650	104	21	[25]

^a^Meat, milk and egg have been considered as the total animal products.

## Discussion

Studying the antibiotic consumption pattern in animals in the present work, revealed that tetracyclines had the highest rate of consumption in the investigated Iranian farms. Furthermore, oxytetracyclin was intensively prescribed for both livestock and poultry followed by gentamycin and tylosin in livestock and furofenocole and sulfodiazin in poultry.

Tetracyclines are broad-spectrum and the most commonly used antibiotics in many countries mainly for prophylactic and growth promotion purpose. It was reported that in Britain, tetracycline, sulfonamides and macrolids formed 90% of the total used livestock antibiotics by 61%, 19% and 9%, respectability, whilst the corresponding figure in South Korea was reported to be 45%, 15% and 4%, respectively [24]. The findings of the present study on the antibiotic consumption index for some countries were in consistent with the previous reports [13,15,26]. It can be inferred that the lack of good husbandry practice (GHP) in livestock and poultry farms has led to a higher consumption of wide-spectrum antibiotic in Iran. As shown in Table 5, the antibiotic factor in Iran is lower than South Korea but more than other countries. Considering annual meat production, in Iran, antibiotic consumption factor is counted as 695 mg/kg, while in Australia, European and the USA it is reported to be 27,104 and 300 mg/kg, respectively. It means that antibiotic consumption factor in Iran is 25, 6.6 and 2.3 times more than that of Australia, Europe and the USA, respectively.

Exposure evaluation of a food contaminant is usually carried out by measuring the residues in food samples. When antibiotic residue in animal originated food comes in to consideration as a potential human hazard, it needs to perform a study in a very large size. In addition, elaborated laboratory techniques are required. Alternatively, estimation of per capita exposure to antibiotics using a representative national data is applicable. However, in Iran, official reports do not cover the antibiotics utilized in the animal husbandry. Therefore, in the presented study, such information was provided via correspondence from the veterinary organization. Determination of the type and the quantity of antibiotic consumption at the farm level is not mainly feasible due to farmers’ economic problems. Antibiotic consumption factor is considered as a general index for risk assessment of exposure to antibiotic residues [24]. Based on the results of a study, 100 mg antibiotic substances is consumed in animal husbandry per unite weight (kg) of produced meat in Europe. Such data was reported to be 26 mg in Australia [10,12].

In line with the results of present study, recent experimental studies in this country, carried out on the antibiotic residues in the food animals highlighted a high violation rate among the tested samples; in a couple of studies 14 to 24% of the raw milk samples were detected to be positive in terms of antibiotic residues [13,14]. In addition, a market survey showed that up to 17.6% of the chickens specimens (liver, kidney and mussel) were contaminated with chloramphencol residues [15]. In another study, 60 percent of the chiken meat samples were shown to have tetracyclin residue of which 10 percent exeeded the regulated limit (100 μg/kg) [27]. Also, conatamination of raw as well as processed bovin milk samples with the high levels of tertacyclin residues were observed in a few studies [16,28,29].

Based on the information presented in Table 2. in 2010, over 1806 tons of antibiotic active substances were consumed in livestock and poultry farms in Iran of which 66.4% was used in cattle farms. Considering the annual production of meat, milk and egg, the antibiotic consumption index was 133 mg/kg; Note that if meat annual production comes in to consideration, the antibiotic index will increase to 695 mg/kg.

It has been reported that annually thousands ton of antibiotics were used in the world. The United States of America with the annual consumption of 11148 tons is the highest consumer of livestock antibiotic worldwide [24]. According to an official report, 78% of a total of 15890 ton antibiotic substances used in US was specified for non- therapeutic agricultural purpose [30]. However, because of the higher animal production rate, the antibiotic consumption index seems to be less than that of Iran.

Previous studies have shown that a direct relation exists between the long-term antibiotic consumption in a society and the prevalence of antibiotic resistant bacteria [6,11]. According to the results of the present study, for instance, antibiotic consumption factor in South Korea was counted as the highest (728.6 mg/kg) among the investigated countries. This may be associated with the high prevalence of antibiotic resistant strains, isolated from livestock in Korea [31]. In addition, a high level of antibiotic consumption factor in Iran, reported in this study, might explain the development of antibiotic resistance among microbial strains isolated from animal-originated foods in several reports in Iran [32]. In a study carried out in a western part of Iran, *Escherichia coli* and *Streptococcus* strains involved in mastitis infection were positive for antibiotic resistance; 52-84% of *E. coli* isolates and 13-20% of *Streptococcus* strains were found to be resistant to penicillin, oxy-tetracycline, streptomycin, erythromycin and colistin [33]. Moreover, there is also substantial evidence on the existence of antibiotic resistant bacteria from human origin in Iran [34,35]. This could be partly due to the long-term intake of antibiotic residues via animal products. Such issues may become more complicated in future if the national supervision and inspection are not implicated. Factors such as improvement of monitoring system on the distribution and consumption of livestock specified antibiotics in the community, implementation of Good Husbandry Practices (GHP) in the farms, progress in farmers educational programs can be helpful in this regards.

Focusing on the farms located in a specific geographical area could be regarded as a limitation in this study and it is necessary to perform a more comprehensive study in future. The heterogeneous nature of previous research works on the exposure assessment of antibiotics through foods in Iran makes it difficult to reach to a precise conclusion in the current situation of public exposure to antibiotics in Iran.

## Conclusion

The present study shows that in Iran, antibiotic consumption in veterinary is noticeable and the consumption index is more than the developed countries. The qualitative pattern of antibiotic consumption in both cattle and poultry farms almost resembled that of other countries in which bacteriostatic antibiotics such as tetracycline has the highest share. However, their consumption found to be good among other countries. It seemed that the risk assessment of exposure to this class of antibiotics is an urgent need in Iran.

## Competing interests

The authors declare that they have no competing interests.

## Authors’ contributions

FA carried out the present survey as the first part of his MSc thesis dissertation in Food Safety and drafted the manuscript. MM supervised the project and participated in review and correction of the manuscript. MJ contributed in the correction of the final draft of the manuscript. All authors read and approved the final manuscript.
